# Safety and Efficacy of Sequential Labor Induction Methods After Failed Primary Induction of Labor: A Retrospective Cohort Study

**DOI:** 10.7759/cureus.112216

**Published:** 2026-07-07

**Authors:** Noureen Waleem, Neeru Arora, Muna Al Badi, Houda N Al Yaqoobi, Farah Khan, Rashid Sultan Al Muqbali

**Affiliations:** 1 Department of Obstetrics and Gynecology, Ministry of Health, Ibri Regional Hospital, Ibri, OMN; 2 Department of Obstetrics and Gynecology, Hamdard University Hospital, Karachi, PAK; 3 Department of Medicine, Ministry of Health, Ibri Regional Hospital, Ibri, OMN

**Keywords:** caesarean section, cervical ripening, dinoprostone, foley catheter, induction of labour, labor induction outcome, maternal outcome, neonatal outcomes, prostaglandin e2, sequential induction

## Abstract

Background: Induction of labor (IOL) is a common obstetric intervention; however, management following failed primary induction remains clinically challenging. Sequential use of pharmacological and mechanical induction methods is practiced in many institutions, although evidence supporting this approach remains limited.

Objective: To evaluate the safety and effectiveness of sequential induction strategies using prostaglandin E_2_ (PGE_2_) gel, dinoprostone vaginal pessary (Propess), and intracervical Foley catheter following failed primary induction.

Materials and methods: This retrospective cohort study was conducted at Ibri Regional Hospital in Oman between January 2023 and December 2025. Women with singleton cephalic pregnancies at ≥37 weeks' gestation who failed an initial induction course and subsequently underwent sequential induction were included. Participants were categorized into six groups (A-F) according to the induction sequence used. Group F (n = 1) was included in the overall cohort but was reported descriptively and excluded from inferential analyses because of the single-case limitation. The primary outcome was vaginal delivery. Secondary outcomes included achievement of active labor, induction-to-delivery interval, mode of delivery, maternal complications, and neonatal outcomes. Statistical analyses were performed using IBM SPSS Statistics version 29.0 (IBM Corp., Armonk, New York, USA). The Kruskal-Wallis test and chi-square test were used for group comparisons, with p < 0.05 considered statistically significant.

Results: A total of 145 women underwent sequential induction. Among groups A-E, 125 women (86.8%) achieved active labor, and 96 (66.7%) had a spontaneous vaginal delivery. Rates of progression to active labor did not differ significantly among groups A-E (p = 0.353). Among women who achieved active labor, vaginal delivery rates differed significantly according to the induction sequence used (p < 0.001), with the highest rate observed in the repeated PGE_2_ gel group. However, this group also had a significantly higher parity. The induction-to-delivery interval differed significantly between groups (p < 0.001), with shorter intervals observed among women receiving prostaglandin followed by an intracervical Foley catheter. Serious maternal complications were uncommon, and no cases of uterine rupture or placental abruption were recorded. Neonatal outcomes, including Apgar scores and neonatal intensive care unit (NICU) admissions, were comparable across groups.

Conclusion: Sequential induction following failed primary IOL was associated with acceptable maternal and neonatal outcomes in the study cohort, and the strategies involving dinoprostone formulations may be associated with a reduced likelihood of cesarean delivery in selected patients. Prospective studies with standardized protocols and multivariable adjustment are required to determine the optimal sequencing strategy.

## Introduction

Induction of labor (IOL) is one of the most commonly performed obstetric interventions, occurring in approximately 20-30% of all pregnancies worldwide, with its frequency increasing over recent decades [1]. Common indications include gestational diabetes mellitus, hypertensive disorders of pregnancy, fetal growth restriction, reduced fetal movements, post-term pregnancy, and prelabor rupture of membranes. The success of labor induction is significantly influenced by baseline cervical status, commonly assessed using the Bishop score, a standardized tool for predicting induction outcomes [2].

The safety of labor induction is evaluated across both maternal and neonatal domains. Key maternal complications include uterine hyperstimulation, postpartum hemorrhage, chorioamnionitis, and, in rare cases, uterine rupture. Neonatal condition at birth is routinely assessed using the Apgar scoring system, which assigns scores at one and five minutes after birth to evaluate the newborn's physiological status [3]. An unfavorable cervical status has been consistently associated with prolonged induction, failed induction, and increased cesarean delivery rates [4].

Both pharmacological agents, including prostaglandin E_2_ gel and dinoprostone vaginal pessary, and mechanical methods, such as intracervical balloon catheters, are routinely used for cervical ripening. Mechanical methods are generally associated with lower rates of uterine tachysystole and hyperstimulation than prostaglandins, while maintaining comparable induction efficacy [5-10].

Management after failed primary induction remains one of the most clinically challenging scenarios in obstetric practice. Clinicians may proceed directly to cesarean section or attempt a sequential induction strategy using an alternative pharmacological or mechanical method. The safety profile of sequential strategies is of particular concern, as repeated uterotonic exposure may theoretically increase the risk of uterine hyperstimulation, maternal infection, and hemorrhage in addition to prolonging fetal exposure to an unfavorable intrauterine environment. Despite the widespread use of sequential induction strategies in routine obstetric practice, evidence comparing different sequencing approaches remains scarce, and major international guidelines offer limited guidance on the optimal management of failed primary induction [11-13].

At Ibri Regional Hospital, sequential induction approaches are commonly employed after failed primary induction, including switching between dinoprostone formulations, repeating prostaglandin administration, or transitioning from pharmacological to mechanical methods. Accordingly, this study aimed to evaluate the effectiveness and safety of sequential induction strategies following failed primary induction. Maternal and neonatal outcomes were compared across different combinations of pharmacological and mechanical induction methods. The findings may contribute to the development of evidence-based local protocols and inform future prospective research.

## Materials and methods

Study design and setting

This retrospective cohort study was conducted in the Department of Obstetrics and Gynecology at Ibri Regional Hospital, Oman, between January 2023 and December 2025. Ethics approval was obtained from the Institutional Review Committee of Ibri Regional Hospital (approval no. 29953). Patient confidentiality was maintained throughout the study, and all data were anonymized in accordance with the Declaration of Helsinki.

Study population

Women with singleton cephalic pregnancies at ≥37 weeks' gestation who underwent sequential induction after failed primary induction were included. Failed primary induction was defined as failure to achieve active labor (cervical dilation ≥5 cm with regular uterine contractions) following completion of a full initial induction course, consistent with the institutional protocol. The initial course consisted of either dinoprostone gel (PGE_2_ gel/Prostin E_2_), administered at up to three doses at eight-hour intervals; a controlled-release dinoprostone vaginal pessary (Propess, 10 mg), used for up to 24 hours; or intracervical Foley catheter insertion according to institutional protocol. Only women with a reassuring fetal cardiotocography at reassessment were considered eligible.

Inclusion criteria were singleton pregnancy, cephalic presentation, gestational age ≥37 weeks, confirmed failed primary induction, and no contraindication to vaginal delivery. Women were excluded if they had a previous uterine scar, non-cephalic presentation, intrauterine fetal demise, major fetal anomaly, or a known hypersensitivity to induction agents.

**Figure 1:**
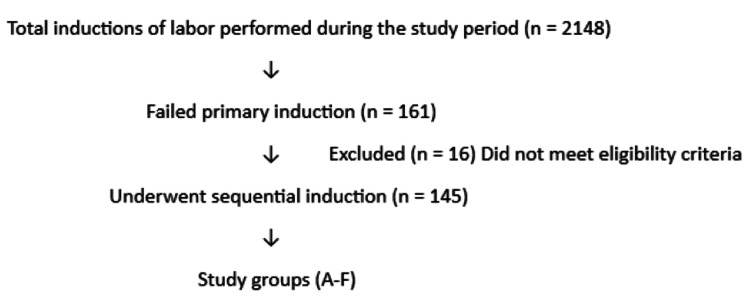
Patient selection flow diagram.

During the study period, 2,148 inductions of labor were performed. Among these, 161 women met the definition of failed primary induction. Of these, 145 women underwent sequential induction and met the inclusion criteria, forming the final study cohort. The remaining 16 women were excluded because of incomplete records or failure to meet eligibility criteria. The patient selection process is illustrated in Figure 1.

Sequential induction groups

Women were categorized into six groups (A-F) according to the induction sequence used, as shown in Table 1. This table was compiled by the authors of the present study based on the sequential induction regimens used at Ibri Regional Hospital. Group C included women who transitioned from either dinoprostone gel or a dinoprostone vaginal pessary to an intracervical Foley catheter. These sequences were combined because both represented a pharmacological-to-mechanical induction strategy, and the individual subgroup sizes were insufficient for independent analysis. Group D comprised women who required three or more sequential induction courses using mixed induction methods and therefore represented a heterogeneous subgroup. Consequently, this group was analyzed primarily descriptively. Group F (n = 1) was included in the total cohort but is reported descriptively only and excluded from inferential analysis.

**Table 1:** Classification of sequential induction groups. This table was compiled by the authors of the present study from the sequential induction regimens used at Ibri Regional Hospital. PG Gel: dinoprostone gel (Prostin E_2_); Propess: controlled-release dinoprostone vaginal insert (10 mg).

Group	n	First course	Second course	Third course
A	8	PG Gel (≤3 doses, eight-hourly)	Propess (24 h)	-
B	17	Propess (24 h)	PG Gel (≤3 doses)	-
C	18	Propess or PG Gel	Foley Catheter	-
D	34	Three or more induction courses using mixed sequential methods		
E	67	PG Gel	PG Gel (repeated)	-
F	1	Foley Catheter	PG Gel	-

Data collection

Data were extracted from electronic and paper medical records using a standardized data collection form (see Appendix 1). Variables collected included maternal age, parity, body mass index (BMI), gestational age, indication for induction, Bishop score before induction (assessed according to the original Bishop scoring system [2]), use of oxytocin augmentation, artificial rupture of membranes (ARM), induction-to-delivery interval, mode of delivery, maternal complications, and neonatal outcomes.

Outcomes

The primary outcome was successful vaginal delivery. Secondary outcomes included achievement of active labor, induction-to-delivery interval, cesarean section indication, maternal complications (including postpartum hemorrhage, uterine hyperstimulation, chorioamnionitis, uterine rupture, and placental abruption), and neonatal outcomes (including Apgar scores, umbilical cord pH, birth weight, and neonatal intensive care unit (NICU) admission). Apgar scores were assigned at one and five minutes after birth according to the standardized Apgar scoring system [3]. Neonatal intensive care unit (NICU) admission criteria were based on institutional neonatal assessment protocols and included respiratory distress, glycemic instability, congenital anomaly evaluation, or cardiorespiratory monitoring.

Statistical analysis

Statistical analysis was performed using IBM SPSS Statistics for Windows, version 29.0 (IBM Corp., Armonk, New York, USA). Continuous variables were summarized as mean ± standard deviation or median with interquartile range according to data distribution. Categorical variables were presented as frequencies and percentages. Normality of continuous variables was assessed using the Shapiro-Wilk test. Group comparisons for continuous variables were performed using the Kruskal-Wallis test, as several variables demonstrated non-normal distribution. Categorical variables were compared using chi-square or Fisher's exact test where appropriate. Due to limited subgroup sample sizes, multivariable regression analysis was not feasible. The study was therefore considered exploratory. Findings regarding comparative efficacy between groups should be interpreted cautiously because of potential confounding, particularly by parity. A p-value of <0.05 was considered statistically significant.

## Results

Baseline characteristics

A total of 145 women were included in the analysis and classified into six sequential induction groups (A-F) according to the induction regimen used (Table 1). One woman in Group F was included in the overall cohort but was reported descriptively because inferential analysis was not possible. The most common indications for induction were gestational diabetes mellitus, intrauterine growth restriction, and reduced fetal movements. There were no statistically significant differences between groups (A-E) in mean gestational age, BMI, or initial Bishop score (p = 0.976, p = 0.224, and p = 0.152, respectively). However, maternal age (p = 0.018) and parity (p < 0.001) differed significantly across groups (A-E). Women in Group E had the highest median parity (Table 2).

**Table 2:** Baseline characteristics by induction group (A-E). Group F (n = 1) is excluded from this table as it was not included in inferential group comparisons. BMI: body mass index.

Variable	Group A (n = 8)	Group B (n = 17)	Group C (n = 18)	Group D (n = 34)	Group E (n = 67)	p-value
Mean age (years)	33.6	31.8	28.8	31.0	32.1	0.018
Median parity	0.5	1.0	0	1.0	2.0	<0.001
Mean gestational age (weeks)	38.4	38.5	38.4	38.3	38.5	0.976
Mean BMI (kg/m²)	30.3	31.2	29.4	33.1	30.6	0.224
Initial Bishop score (mean)	1.3	2.0	1.6	1.1	1.8	0.152

Oxytocin augmentation and artificial rupture of membranes

Overall, 66 of 144 women (45.8%) received oxytocin augmentation (Table 3). The requirement for oxytocin differed significantly across Groups A-E (p = 0.015), with the lowest use in Group E (31.3%) and the highest proportions in Groups C and A (66.7% and 62.5%, respectively). Artificial rupture of membranes (ARM) was performed in 98 women (68.1%) and also differed significantly across Groups A-E (p < 0.001), being most frequent in Group C (94.4%) and least frequent in Groups A and B (25.0% and 35.3%, respectively).

**Table 3:** Oxytocin augmentation and artificial rupture of membranes (ARM) use by group. ARM: artificial rupture of membranes.

Group	Oxytocin: Yes n (%)	Oxytocin: No n (%)	ARM: Yes n (%)	ARM: No n (%)
A (n = 8)	5 (62.5%)	3 (37.5%)	2 (25.0%)	6 (75.0%)
B (n = 17)	7 (41.2%)	10 (58.8%)	6 (35.3%)	11 (64.7%)
C (n = 18)	12 (66.7%)	6 (33.3%)	17 (94.4%)	1 (5.6%)
D (n = 34)	21 (61.8%)	13 (38.2%)	24 (70.6%)	10 (29.4%)
E (n = 67)	21 (31.3%)	46 (68.7%)	49 (73.1%)	18 (26.9%)
p-value	0.015		<0.001	

Achievement of active labor

Of the 144 women in groups (A-E), 125 (86.8%) reached active labor (cervical dilation ≥5 cm with regular contractions). All women in Group C reached active labor, compared with 88.2% in Group D, 86.6% in Group E, 76.5% in Group B, and 75.0% in Group A. These differences were not statistically significant across groups A-E (p = 0.353; Table 4). Among the 19 women (13.2%) who did not reach active labor, almost all underwent cesarean section (17/19); two women in Groups D and E delivered vaginally after rapid cervical progression, one of whom developed tachysystole following ARM.

**Table 4:** Achievement of active labor by group. *Group F (n = 1) was reported as descriptive only and excluded from inferential comparisons.

Group	Reached active labor n (%)	Did not reach n (%)	Total
A	6 (75.0%)	2 (25.0%)	8
B	13 (76.5%)	4 (23.5%)	17
C	18 (100.0%)	0 (0.0%)	18
D	30 (88.2%)	4 (11.8%)	34
E	58 (86.6%)	9 (13.4%)	67
F*	1 (100.0%)	0 (0.0%)	1
Total (p = 0.353) (A-E)	125 (86.8%)	19 (13.2%)	144

Mode of delivery

Among the 144 women in groups (A-E), 96 (66.7%) achieved spontaneous vaginal delivery, 2 (1.4%) had vacuum-assisted delivery, and 46 (31.9%) underwent lower segment cesarean section (LSCS) (Table 5). The distribution of mode of delivery across groups (A-E) did not differ significantly (p = 0.128). Group E had the highest proportion of spontaneous vaginal delivery (80.6%) and the lowest LSCS rate (17.9%), whereas Group B had the highest cesarean rate (52.9%). Group F (n = 1) was reported as descriptive only and excluded from inferential comparisons. 

Among women in groups (A-E) who achieved active labor (n = 125), vaginal delivery rates differed significantly between induction sequences (p < 0.001). Group E achieved the highest vaginal delivery rate among those in active labor (93.1%; 54/58), compared with 61.5% (8/13) in Group B, 55.6% (10/18) in Group C, and 60.0% (18/30) in Group D.

**Table 5:** Mode of delivery and vaginal delivery success among women who reached active labor, by group. SVD: spontaneous vaginal delivery; LSCS: lower segment cesarean section *Group F (n = 1) was reported as descriptive only and excluded from inferential comparisons.

Group	SVD n (%)	Vacuum n (%)	LSCS n (%)	Total	Vaginal delivery among those in active labor
A	6 (75.0%)	0 (0.0%)	2 (25.0%)	8	6/6 (100.0%)
B	8 (47.1%)	0 (0.0%)	9 (52.9%)	17	8/13 (61.5%)
C	10 (55.6%)	0 (0.0%)	8 (44.4%)	18	10/18 (55.6%)
D	18 (52.9%)	1 (2.9%)	15 (44.1%)	34	18/30 (60.0%)
E	54 (80.6%)	1 (1.5%)	12 (17.9%)	67	54/58 (93.1%)
F*	1 (100.0%)	0 (0.0%)	0 (0.0%)	1	1/1 (100.0%)
A-E	96 (66.7%)	2 (1.4%)	46 (31.9%)	144	-
p-value (A-E)	0.128				<0.001 (active labor subset)

Indications for cesarean section

Among the 46 women who underwent lower segment cesarean section (LSCS), fetal distress was the leading indication, accounting for 23 cases (50.0%), followed by failed IOL in 8 (17.4%), non-progress of labor in 7 (15.2%), maternal request in 7 (15.2%), and cord presentation in 1 (2.2%) (Table 6). Group D contributed the highest number of cesarean deliveries (15/46, 32.6%), followed by Group E (12/46, 26.1%), Group B (9/46, 19.6%), Group C (8/46, 17.4%), and Group A (2/46, 4.3%). The distribution of cesarean indications across groups was not statistically different (p = 0.609). Among the 28 women who underwent LSCS after reaching active labor, fetal distress remained the leading indication (64.3%), followed by non-progress of labor (25.0%), maternal request (7.1%), and cord presentation (3.6%).

**Table 6:** Indications for LSCS by group. Percentages within each group column reflect the proportion of each LSCS indication relative to total LSCS cases within that group. LSCS: lower segment cesarean section; IOL: induction of labor; NPOL: non-progress of labor. Group F had no LSCS and is not included in this table.

LSCS indication	Group A n (%)	Group B n (%)		Group C n (%)	Group D n (%)	Group E n (%)	Total n (%)
Fetal distress	1 (50.0%)		5 (55.6%)	6 (75.0%)	5 (33.3%)	6 (50.0%)	23 (50.0%)
Failed IOL	1 (50.0%)		1 (11.1%)	0 (0.0%)	3 (20.0%)	3 (25.0%)	8 (17.4%)
NPOL	0 (0.0%)		1 (11.1%)	2 (25.0%)	4 (26.7%)	0 (0.0%)	7 (15.2%)
Maternal request	0 (0.0%)		2 (22.2%)	0 (0.0%)	2 (13.3%)	3 (25.0%)	7 (15.2%)
Cord presentation	0 (0.0%)		0 (0.0%)	0 (0.0%)	1 (6.7%)	0 (0.0%)	1 (2.2%)
Total	2 (100.0%)		9 (100.0%)	8 (100.0%)	15 (100.0%)	12 (100.0%)	46 (100%)

Induction-to-delivery interval

Induction-to-delivery intervals differed significantly across groups (p < 0.001; Table 7). Group D, which comprised women requiring three or more sequential induction courses, had the longest median induction-to-delivery interval at 97.9 hours. Among the two-course regimens, Group C (prostaglandin followed by a Foley catheter) had the shortest median interval (60.1 hours), followed by Groups B (65.5 hours) and E (66.4 hours). Group F had the shortest interval overall (38.6 hours), but this observation was based on a single case and should be interpreted with caution.

**Table 7:** Induction-to-delivery interval by group. n values reflect the number of women who achieved vaginal delivery and for whom the induction-to-delivery interval could be calculated; women who underwent LSCS are not included in the interval calculation. Induction-to-delivery interval calculated from the start of the first sequential induction agent to delivery. p < 0.001 across groups A-E (Kruskal-Wallis test). *Group F: single case only; descriptive statistics only.

Group	n	Mean (h)	SD	Median (h)	Q1 (h)	Q3 (h)	Min-Max (h)
A	6	73.1	15.0	71.9	60.1	84.2	57.1-92.9
B	8	73.0	18.6	65.5	60.8	76.1	58.0-109.9
C	10	61.8	8.7	60.1	58.8	68.0	45.9-74.9
D	19	95.8	21.2	97.9	83.3	102.9	48.6-132.4
E	55	71.8	16.9	66.4	60.8	82.3	44.9-112.6
F*	1	38.7	-	38.6	-	-	38.6
p-value	(p < 0.001) across groups A-E						

Maternal outcomes

Serious maternal complications across groups (A-E) were rare (Table 8). One woman (0.7%, p = 0.660) developed tachysystole following ARM, while no cases of uterine hyperstimulation, uterine rupture, cervical tear, or placental abruption were recorded. One woman (0.7%) in Group D developed a maternal infection (p = 0.660 across groups), and three women (2.1%, p = 0.366) experienced postpartum hemorrhage (two in Group E and one in Group C). 

**Table 8:** Maternal complications by group. Group F (n = 1) is excluded from comparative p-value calculations.

Complication	A	B	C	D	E	Total	p-value
Uterine hyperstimulation	0	0	0	0	0	0	Not estimable
Tachysystole	0	0	0	0	1	1 (0.7%)	p = 0.660
Uterine rupture/cervical tear	0	0	0	0	0	0	Not estimable
Abruptio placentae	0	0	0	0	0	0	Not estimable
Maternal infection	0	0	0	1	0	1 (0.7%)	p = 0.660
Postpartum hemorrhage	0	0	1	0	2	3 (2.1%)	p = 0.366

Neonatal outcomes

Neonatal outcomes were generally favorable and did not differ significantly across groups A-E (all p > 0.05; Table 9). The mean birth weight was 3000.5 g (median 3010 g; range 1810-3845 g), and 15 neonates (10.4%) weighed less than 2500 g; no infant had a very low birth weight (<1500 g). Seven neonates (4.9%) had a first-minute Apgar score ≤7, including one neonate (0.7%) with a score <7. All five-minute Apgar scores were ≥7. Umbilical cord pH was available in 19 cases (13.2%), with a mean of 7.276; 2 neonates (10.5% of those with values) had a cord pH < 7.2. NICU admission was required in 11 neonates (7.6%), predominantly for indications such as congenital anomaly evaluation, glycemic monitoring, or cardiorespiratory observation rather than intrapartum complications.

**Table 9:** Neonatal outcomes. Group F (n = 1) is excluded from comparative p-value calculations. p-values from Kruskal-Wallis or chi-square test across groups A-E.

Neonatal outcome	Result	p-value (across groups)
Mean birth weight (g)	3000.5 (SD 362; median 3010; range 1810-3845 g)	0.235
Birth weight <2500 g n (%)	15 (10.4%)	-
Sex ratio (female: male)	74:71	0.590
First Apgar score (mean)	8.84 (range 4-10)	0.734
First Apgar ≤7 n (%)	7 (4.9%)	-
First Apgar <7 n (%)	1 (0.7%)	-
Second Apgar score (mean)	9.92	0.676
Second Apgar <7 n (%)	0 (0.0%)	-
Cord pH (n = 19; mean)	7.276 (range 7.14-7.40)	0.438
Cord pH <7.2 n (%)	2/19 (10.5%)	-
NICU admission n (%)	11 (7.6%)	0.129

## Discussion

This study examined different induction sequences in 145 women who failed primary IOL at a regional hospital in Oman. After exclusion of Group F from inferential analyses, the overall vaginal delivery rate among Groups A-E was 66.7%. The absence of uterine rupture or placental abruption suggests that sequential induction following failed primary induction may be a feasible and safe strategy in this setting. The cesarean delivery rate of 31.9% is consistent with the higher baseline risk expected in a cohort that had already failed an initial induction attempt and compares favorably with rates reported in similar retrospective studies [11,12].

The absence of a statistically significant difference in overall mode of delivery between groups (p = 0.128) does not imply that all sequences are equivalent. Among women who reached active labor, vaginal delivery rates differed significantly (p < 0.001), with Group E (repeated PGE_2_ gel) demonstrating the highest rate at 93.1%. However, this group also had the highest median parity, and parity is a well-established predictor of vaginal delivery success, particularly once cervical ripening has commenced. It is therefore likely that the favorable outcomes in Group E reflect, at least in part, the confounding effect of higher parity rather than a specific advantage of the repeated PGE_2_ gel regimen, a limitation inherent to the non-randomized design of this study. Consequently, the apparent higher vaginal delivery rate observed in Group E should not be interpreted as evidence of treatment superiority. These findings highlight the importance of parity adjustment in future comparative analyses of different induction sequences [13].

The induction-to-delivery interval was significantly longer in Group D (median 97.9 hours), as expected, because these women required three or more sequential induction courses and represented the most refractory cases in the cohort. Among the two-course regimens, Group C (prostaglandin followed by a Foley catheter) had the shortest median interval at 60.1 hours. This suggests that transitioning from pharmacological to mechanical methods after failed induction may be associated with a shorter induction-to-delivery interval. Mechanical methods, such as the Foley catheter, are believed to act through combined mechanical dilation and stimulation of endogenous prostaglandin release and are broadly comparable to pharmacological agents in overall induction efficacy while carrying a lower risk of uterine hyperstimulation [8,9]. These findings are consistent with current WHO recommendations supporting the inclusion of mechanical methods as a core component of induction protocols [14].

Groups A and B provide an indirect comparison between the two PGE_2_ formulations. Group B (Propess first, then gel) had a shorter median induction-to-delivery interval than Group A (gel first, then Propess). Although these groups are small and the comparison should be treated with caution, this observation is consistent with published data indicating that Propess, as a sustained-release dinoprostone formulation, tends to achieve more efficient cervical ripening and shorter induction intervals than the gel formulation [15-18]. This may reflect the pharmacokinetic advantage of sustained prostaglandin delivery in promoting progressive cervical change.

The role of the Foley catheter as a rescue mechanical method is further supported by the induction interval data from Group C. In the present cohort, only one woman developed tachysystole, and no uterine hyperstimulation events required emergency intervention. This aligns with the favorable safety profile of sequential induction regimens reported in the existing literature, where the risk of serious maternal complications with combined pharmacological and mechanical approaches has consistently been reported as low [11-13]. Systematic reviews and randomized controlled trials have further confirmed that the Foley catheter is broadly comparable to prostaglandins in overall induction efficacy while offering a significantly lower risk of uterine hyperstimulation, making it a particularly valuable rescue option in the sequential induction setting [9,19].

The maternal safety profile observed in this cohort was acceptable. No cases of uterine rupture, cervical tear, or placental abruption were recorded across any group, which is clinically significant given that repeated uterotonic exposure in sequential protocols is sometimes cited as a theoretical concern [5]. Postpartum hemorrhage occurred in three women (2.1%), and one case of maternal infection was recorded (0.7%). The low frequency of maternal complications, including the absence of uterine hyperstimulation requiring emergency intervention, is consistent with previously reported safety data for sequential induction approaches and with the generally lower hyperstimulation risk associated with mechanical methods [1,6,10-12]. Only one woman developed tachysystole, which resolved without emergency intervention. Coste-Mazeau et al. reported acceptable maternal safety outcomes with repeated use of the dinoprostone vaginal insert in the multicenter RE-DINO randomized trial [13], which is broadly consistent with the low complication rates observed in the present cohort, although differences in study design and patient selection limit direct comparison.

Neonatal outcomes were reassuring across all groups. Apgar scores at one and five minutes were within normal ranges in the vast majority of neonates, and all second Apgar scores were ≥7. Most NICU admissions were attributable to neonatal observation or pre-existing clinical concerns, including glycemic monitoring, congenital anomaly evaluation, and cardiorespiratory observation, rather than intrapartum complications. There were no significant differences between groups in Apgar scores, umbilical cord pH, or NICU admission rates. These findings are consistent with the broader literature demonstrating that sequential induction strategies, when conducted with appropriate fetal monitoring, do not compromise neonatal safety [11-13].

This study has several limitations. It was retrospective, single-center, and non-randomized, so confounding by indication is likely, as regimen assignment was based on clinical judgment rather than a standardized protocol. Baseline differences between groups, particularly the higher parity observed in Group E, may have influenced labor progression and mode of delivery, but these factors could not be fully adjusted for because of the limited sample size. Group D was heterogeneous by design, which limits the interpretation of regimen-specific effects. Umbilical cord pH data were available in only a minority of cases, and the small sizes of Groups A, B, and F limit subgroup analysis. Comparative studies evaluating double-balloon catheter regimens and misoprostol-based sequences are needed to expand the evidence base [20]. Future prospective studies with larger, balanced groups and multivariable adjustment are required to define the optimal sequence of induction methods [21,22].

## Conclusions

Sequential induction after failed primary labor induction was associated with acceptable maternal and neonatal outcomes in this cohort. Most women avoided cesarean delivery, with two-thirds achieving vaginal birth. No cases of uterine rupture or placental abruption were recorded. Among the induction sequences evaluated, prostaglandin-based regimens and the use of a Foley catheter as a rescue mechanical method were associated with favorable outcomes, particularly regarding the induction-to-delivery interval; however, these observations should be interpreted with caution because of potential confounding. These findings may inform the development of a structured local stepwise induction protocol tailored to parity, Bishop score, and clinical status. However, as a single-center retrospective study, these findings are exploratory and require confirmation through larger prospective studies with multivariable adjustment to determine the optimal induction sequence.

## Appendices

Appendix 1 

**Table 10:** Standardized data collection form used for retrospective data extraction. GDM: gestational diabetes mellitus; IUGR: intrauterine growth restriction; FM: fetal movements; PROM: prelabor rupture of membranes; ARM: artificial rupture of membranes; SVD: spontaneous vaginal delivery; LSCS: lower segment cesarean section; IOL: induction of labor; NPOL: non-progress of labor; NICU: neonatal intensive care unit. *The Bishop score was assessed using the original Bishop scoring system [2]. **Apgar scores assigned using the standardized Apgar scoring system [3].

Domain	Variable	Data type/description
Maternal demographics	Age	Years (continuous)
	Parity	Number of previous deliveries
	Body mass index (BMI)	kg/m² (continuous)
Obstetric details	Gestational age	Weeks (continuous)
	Indication for induction	Categorical (GDM, IUGR, reduced FM, hypertension, post-term, PROM, other)
	Initial Bishop score	Numeric score 0-13 (Bishop scoring system) *
Induction details	Primary induction agent	PG Gel/Propess/Foley catheter
	Sequential induction group	A/B/C/D/E/F
	Oxytocin augmentation	Yes/No
	Artificial rupture of membranes (ARM)	Yes/No
	Induction-to-delivery interval	Hours (continuous)
Delivery outcomes	Mode of delivery	SVD/Vacuum/LSCS
	Indication for LSCS (if applicable)	Fetal distress/Failed IOL/NPOL/Maternal request/Cord presentation
Maternal complications	Postpartum hemorrhage	Yes/No
	Uterine hyperstimulation/tachysystole	Yes/No
	Maternal infection (chorioamnionitis)	Yes/No
	Uterine rupture/cervical tear	Yes/No
	Placental abruption	Yes/No
Neonatal outcomes	Birth weight	Grams (continuous)
	Sex	Female/Male
	Apgar score at 1 minute	0-10 (Apgar scoring system)**
	Apgar score at 5 minutes	0-10
	Umbilical cord pH	Numeric (where available)
	NICU admission	Yes/No (indication if applicable)
